# Modifiable Risk Factors for Dementia in Older Patients Receiving Primary Care: A Retrospective Observational Study

**DOI:** 10.7759/cureus.115704

**Published:** 2026-09-03

**Authors:** Senada Selmanovic, Amila Vujadinovic, Jasna Vasilj Bojic, Danijel Gajic, Emina Bajric Custo, Jasmina Klebic, Munevera Becarevic, Sabina Ademovic Dzambic, Branislav Milic

**Affiliations:** 1 Department of Family Medicine, Public Health Teaching Institution Health Center "Dr. Mustafa Šehović" Tuzla, Faculty of Medicine, University of Tuzla, Tuzla, BIH; 2 Department of Family Medicine, Public Health Teaching Institution Health Center "Dr. Mustafa Šehović" Tuzla, Tuzla, BIH; 3 Department of Family Medicine, Health Center Živinice, Živinice, BIH; 4 Department of Family Medicine, Health Center Orašje, Orašje, BIH; 5 Department of Family Medicine, Health Center Lukavac, Lukavac, BIH; 6 Center for Early Childhood Development, Public Health Teaching Institution Health Center "Dr. Mustafa Šehović" Tuzla, Tuzla, BIH; 7 Department of Family Medicine, Public Health Institution Health Center Banovići, Faculty of Medicine, University of Tuzla, Banovići, BIH; 8 Department of Psychiatry, Health Center Orašje, Orašje, BIH

**Keywords:** cognitive decline, dementia, geriatric medicine, primary health care, risk factors

## Abstract

Background: Dementia represents a major global public health challenge in aging populations, requiring effective primary care identification and prevention strategies. This study evaluated the prevalence and clinical significance of 11 modifiable risk factors among older adults within a primary healthcare setting.

Methods: An observational, retrospective-analytical study was conducted in January 2025 within a family medicine team panel (N = 1122). Participants aged 65 and older were divided into two cohorts: patients with diagnosed dementia (n = 20) and a comparative group without dementia (n = 50). Data on 11 modifiable risk factors were extracted using medical records and clinical anamnesis. Continuous variables were analyzed using Student’s independent samples t-test, while categorical variables were evaluated using Pearson’s chi-square test or Fisher’s exact test (p < 0.05).

Results: Patients with dementia were significantly older (85.50 ± 4.83 vs. 80.34 ± 4.10 years; p < 0.05). Lower educational level was frequent among dementia patients, with 55% (n = 11) having completed primary education compared to 32% (n = 16) in controls (p = 0.160). Obesity (BMI > 30 kg/m²) was significantly less frequent in the dementia group (5%, n = 1 vs. 48%, n = 24; p < 0.05). Arterial hypertension showed a lower prevalence in dementia patients (85%, n = 17 vs. 98%, n = 49; p = 0.038). Hearing impairment was present in all participants (100%). In late life, physical inactivity (90%, n = 18 vs. 74%, n = 37; p = 0.141), social isolation (60%, n = 12 vs. 66%, n = 33; p = 0.636), and depression (55%, n = 11 vs. 64%, n = 32; p = 0.485) showed high overall prevalence across both cohorts without statistically significant group differences.

Conclusion: Modifiable risk factors for dementia are highly prevalent among geriatric primary care patients, representing a widespread cumulative burden. Early identification and systematic screening in family medicine are essential to optimize cognitive health management.

## Introduction

Dementia syndrome encompasses a heterogeneous group of disorders characterized by a progressive and significant decline in cognitive functions, thereby impairing an individual's professional, personal, and social functioning. These cognitive functions refer to complex cortical capabilities, including memory, speech, reasoning, planning, behavior, and executive abilities [1].

Cognitive impairment represents a significant public health challenge that requires a multidisciplinary approach to patient care. The term mild cognitive impairment (MCI) is used for patients who do not meet the diagnostic criteria for dementia. MCI often remains unrecognized because it can be masked by symptoms of depression and anxiety, subsequently progressing into dementia [2].

The concept of dementia encompasses several subtypes with distinct pathophysiological mechanisms. It most commonly presents with mixed manifestations during aging but may also include vascular pathology [3].

It is concerning that approximately 50 million people worldwide suffer from dementia. The most common type is Alzheimer's disease, accounting for approximately 60-70% of cases, while vascular dementia has a prevalence of around 20% [4].

A major concern is the financial burden associated with the treatment and care of patients with dementia. According to a 2019 meta-analysis, 61% of individuals with dementia live in low- and middle-income countries, and due to population aging, this number is trending upward. A vast number of cases remain undiagnosed, which could be attributed to the lack of standard diagnostic procedures (dementia biomarkers) in lower-income countries [5].

In developing and resource-limited settings where systemic support is scarce, early awareness of modifiable risk factors and the consistent application of simple, accessible screening tools (such as the Mini-Mental State Examination (MMSE) test) represent a crucial pillar in the early diagnosis and prevention of dementia within primary healthcare settings [6].

According to the Lancet Commission report, targeting 12 modifiable risk factors across different stages of life could prevent or delay up to 40% of all dementia cases worldwide. These factors include lower levels of formal education in early life; hearing impairment, traumatic brain injury, arterial hypertension, excessive alcohol consumption, and obesity in midlife; and smoking, depression, social isolation, physical inactivity, diabetes, and air pollution in late life. The identification and modification of these factors in primary care settings constitute a pivotal pillar for early diagnosis and dementia prevention, given that they can be directly influenced, unlike non-modifiable risks such as age, sex, and genetic predisposition [7].

Research indicates that, in addition to advanced age and female sex, non-modifiable risk is largely determined by a complex genetic architecture; the identification of novel genetic loci sheds light on familial predisposition and key pathophysiological mechanisms, such as neuroinflammation and amyloid accumulation. Naturally, these are unmodifiable factors, but they highlight the need for secondary dementia prevention, namely, screening [8].

Numerous epidemiological studies show that the accumulation of multiple vascular and behavioral risk factors (such as hypertension, smoking, and physical inactivity) exerts an additive effect on accelerating cognitive decline and the development of incident dementia [9].

The objective of this study was to evaluate the prevalence and distribution of 11 modifiable risk factors identified by the Lancet Commission, encompassing metabolic, behavioral, psychosocial, and sensory parameters, among patients aged 65 years and older with and without a diagnosis of dementia within a primary healthcare setting. Specific objectives were to analyze sociodemographic characteristics (age, sex, education, and head trauma), evaluate metabolic and anthropometric markers (hypertension, diabetes, HbA1c, LDL cholesterol, and body mass index), and assess sensory and behavioral profiles (hearing impairment, physical inactivity, depression, smoking, alcohol misuse, and social isolation) to identify significant differences in risk factor frequencies between the cohorts for improving primary care preventive strategies.

## Materials and methods

Study design

This study was designed as an observational, retrospective-analytical study conducted in January 2025 at the Public Health Institution Health Center "Dr. Mustafa Šehović" Tuzla, Bosnia and Herzegovina. The study was based on the analysis and evaluation of documented patient medical records aimed at identifying non-modifiable parameters (age and sex) and 11 modifiable risk factors for dementia, categorized according to the Lancet Commission framework across three life stages [7]. Evaluated factors included lower education in early life (completion of primary education only, <8 years of formal schooling); midlife factors comprising arterial hypertension (documented diagnosis, resting blood pressure > 140/90 mm/Hg, or antihypertensive therapy), obesity (body mass index (BMI) > 30 kg/m²), hearing impairment (documented medical diagnosis, hearing aid use, or patient/informant-reported functional hearing difficulty during consultation), head injury (documented history of traumatic brain injury), and alcohol misuse (documented excessive intake or >14 units/week); and late-life factors consisting of active smoking, depression (documented diagnosis or active antidepressant use), physical inactivity (< 150 minutes of moderate activity per week), social isolation (infrequent social contact, living alone, or documented social withdrawal), and diabetes mellitus (documented diagnosis, fasting glucose > 7.0 mmol/L, or antidiabetic treatment). Within the Lancet Commission model, the air pollution factor was excluded from this analysis, given the retrospective nature of the study in primary care practice where medical records lack systematically documented data on individual exposure, concentrations, and air quality at the micro-residential location of patients prior to and during disease diagnosis.

Study population

The study included 70 participants aged 65 years and older, registered within a family medicine team serving a total patient panel of 1122 individuals. Based on defined criteria, two study groups were established. The group of patients without dementia comprised 50 consecutively selected patients within the specified age range (chosen in sequential order from the medical records) who had no documented diagnosis of dementia or recorded cognitive decline in their medical files. The comparative group consisted of all 20 patients aged 65 and older with an officially documented diagnosis of dementia receiving treatment, representing an established prevalence of 1.8% in the team's total population.

Diagnosis and verification of dementia

Dementia status was identified in accordance with the International Classification of Diseases, Tenth Revision (ICD-10) criteria (codes F00-F03) [10] and verified through the participants' official medical records at the Health Center. Diagnosis was established through comprehensive clinical evaluations, which included specialist documentation from a neurologist and/or psychiatrist, collateral history, neurocognitive screening via the MMSE, and structural neuroimaging (CT or MRI) where applicable.

Sociodemographic data, non-modifiable risk factors, metabolic parameters, behavioral, and sensory factors were extracted from the participants' medical records.

Sample size justification

The sample size was determined based on the total eligible patient population registered within a single family medicine team panel (N = 1122). A complete census approach was applied for the dementia group, including all 20 registered patients aged 65 years and older with a verified diagnosis. To establish a robust comparative group without cognitive decline, 50 consecutively selected patients within the same age bracket were included, resulting in a total sample size of 70 participants (n = 70) with a case-to-control ratio of 1:2.5. Given the observational nature of this primary care chart review, this sample size captures the entire available clinical cohort within the practice panel, thereby minimizing selection bias. However, with n = 20 cases in the dementia cohort, the study remains underpowered to detect significant differences for less common exposures, which is explicitly acknowledged as a study limitation.

Inclusion and exclusion criteria for study participants are presented in Table 1.

**Table 1:** Criteria for participant selection in the study

Category	Inclusion Criteria	Exclusion Criteria
General Criteria	Registration with the family medicine team; age ≥65 years; signed consent form (patient or legal guardian)	Age <65 years; patients not registered with the specified team; refusal to provide consent for participation
Dementia Group	Officially confirmed diagnosis of dementia in medical records; documented use of dementia therapy	Suspected cognitive decline without a confirmed diagnosis; incomplete medical documentation regarding the diagnosis
Non-dementia Group	Sequential selection from medical files (consecutive); preserved cognitive function without a diagnosis of dementia	Presence of diagnosed dementia or cognitive decline; acute severe conditions preventing assessment

Statistical analysis

All collected data were compiled into a dedicated database and subjected to statistical analysis using IBM SPSS Statistics for Windows, Version 26 (Released 2018; IBM Corp., Armonk, New York, United States). The analysis encompassed methods of descriptive and inferential statistics, where continuous variables were expressed as mean values and standard deviations, while categorical variables were presented as absolute frequencies and percentages. The statistical significance of differences between the observed groups was evaluated using the chi-square test for categorical variables and frequencies, and Student’s t-test for independent samples when comparing continuous clinical parameters. A p-value of less than 0.05 was considered statistically significant.

Ethical considerations

The study protocol was approved by the Ethics Committee of the Public Health Institution Health Center "Dr. Mustafa Šehović" Tuzla. Given the retrospective design of the study involving the analysis of pre-existing, anonymized medical records with no direct participant interaction, the Ethics Committee granted a waiver of informed consent. All patient data were fully de-identified prior to analysis to ensure privacy and confidentiality in accordance with the Declaration of Helsinki.

## Results

Within the analyzed sample of 70 participants aged 65 and older, patients without dementia (n = 50) comprised the primary group, while patients diagnosed with dementia (n = 20) served as the comparative group.

The sociodemographic characteristics of both participant groups (age and sex distribution) are presented in Table 2. Patients with dementia were statistically significantly older compared to patients without dementia (p < 0.05), whereas no statistically significant difference was found between the groups regarding sex distribution (p = 0.164).

**Table 2:** Sociodemographic characteristics of the study participants Data are presented as absolute numbers and percentages, n (%) for categorical variables (sex) and as Mean ± Standard Deviation (SD) for continuous variables (age). Differences between categorical variables were analyzed using the chi-square test (x²), while continuous variables were compared using the independent samples t-test. Statistical significance was established at p < 0.05. n: number of participants; SD: standard deviation; p: probability value. *Statistically significant at p<0.05.

Characteristic	Patients with Dementia (n=20)	Patients without Dementia (n=50)	p-value
Age (years), M ± SD	85.50 ± 4.83	80.34 ± 4.10	< 0.05*
Age range (min–max)	77–90	72–89	—
Sex, n (%)			0.164
Male	10 (50%)	16 (32%)	
Female	10 (50%)	34 (68%)	

The distribution of participants according to their educational level was analyzed. In the group of patients without dementia, 32% (n = 16) had primary education, 52% (n = 26) had secondary education, and 16% (n = 8) held a higher (university) degree. In the group of patients with dementia, 55% (n = 11) had completed primary education, 45% (n = 9) had completed secondary education, while none of the patients (0%; n = 0) held a higher (university) degree. No statistically significant correlation was found between the level of education and the presence of dementia.

Among midlife factors, hearing impairment, as a sensory risk factor, was recorded in all participants across both groups (n = 20; 100% vs n = 50; 100%). A statistically significant difference was observed for arterial hypertension (p < 0.05), which showed a higher prevalence in the group without dementia (n = 49; 98% vs. n = 17; 85%), as well as for obesity based on BMI values (p < 0.05). Specifically, the non-dementia group exhibited a significantly higher mean BMI (29.34 ± 4.53 kg/m²) and a higher proportion of obesity (48%; n=24) compared to the dementia group (26.05 ± 2.74 kg/m²) in which 5% were obese. Conversely, no cases of excessive alcohol consumption were recorded in the sample (0%), while a history of head trauma was identified in a minimal number of participants, with no statistically significant difference between the groups (n = 1; 5% in patients with dementia vs. n = 2; 4% in the non-dementia group) (Table 3).

**Table 3:** Modifiable and non-modifiable midlife risk factors for dementia Data are presented as absolute numbers and percentages, n(%). Statistical comparisons between groups were performed using Fisher's exact test (for cells with expected frequencies < 5) or Pearson's Chi-square test (x²). A p-value < 0.05 was considered statistically significant. n: number of participants; BMI: body mass index; p: probability value. *Statistically significant at p < 0.05

Risk factor	Patients with Dementia (n=20)	Patients without Dementia (n=50)	p-value
Hearing impairment, n (%)	20 (100%)	50 (100%)	—
Arterial hypertension, n (%)	17 (85%)	49 (98%)	0.034*
Excessive alcohol consumption, n (%)	0 (0%)	0 (0%)	—
Obesity (BMI>30 kg/m²), n (%)	1 (5%)	24 (48%)	< 0.05*
History of head trauma, n (%)	1 (5%)	2 (4%)	0.0852

In late life, a high prevalence of physical inactivity was observed in both groups, with a higher rate among patients with dementia (n = 18; 90% vs. n = 37; 74%). Conversely, social isolation (n = 33; 66% vs. n = 12; 60%) and depression (n = 32; 64% vs. n = 11; 55%) were more prevalent in participants without dementia. No statistically significant differences were found between the examined groups regarding these parameters. Similarly, no statistically significant differences were identified in the frequency of diabetes, which was more common in participants without dementia (n = 18; 36% vs. n = 6; 30%), or active smoking status, which was recorded in a small percentage exclusively within the non-dementia group (n = 7; 14% vs. n = 0; 0%) (Table 4).

**Table 4:** Late-life risk factors for dementia Data are presented as absolute numbers and percentages, n(%). Statistical comparisons between groups were performed using Fisher's exact test (for cells with expected frequencies < 5) or Pearson's chi-square test (x²). A p-value < 0.05 was considered statistically significant. n: number of participants; p: probability value.

Risk factor	Patients with Dementia (n=20)		Patients without Dementia (n=50)	p-value
Physical inactivity, n (%)	18 (90%)		37 (74%)	0.141
Social isolation, n (%)		12 (60%)	33 (66%)	0.636
Depression, n (%)	11 (55%)		32 (64%)	0.485
Diabetes mellitus, n (%)	6 (30%)		18 (36%)	0.842
Active smoking, n (%)	0 (0%)		7 (14%)	0.055
Air pollution	Not evaluated		Not evaluated	—

A comparison of clinical and laboratory parameters revealed a statistically significant difference in BMI (p = 0.001), where patients without dementia exhibited a higher mean value (29.34 ±4.53 kg/m²). In contrast, HbA1c and LDL cholesterol levels showed no statistically significant differences between the two groups (Table 5).

**Table 5:** Comparison of continuous clinical and laboratory parameters Data are presented as mean ± standard deviation (SD). Differences between the two independent groups were evaluated using the independent samples t-test. Statistical significance was defined as p < 0.05. n: number of participants; BMI: body mass index; HbA1c: glycated hemoglobin; LDL: low-density lipoprotein cholesterol; SD: standard deviation; p: probability value. *Statistically significant at p<0.05.

Parametar	Patients with Dementia (n=20)	Patients without Dementia (n=50)	p-value
BMI (kg/m²), M ± SD	26.05 ± 2.74	29.34 ± 4.53	<0.05*
HbA1c (%), M ± SD	7.15 ± 0.69	7.13 ± 0.82	0.92
LDL (mmol/L), M ± SD	2.64 ± 0.74	2.72 ± 0.98	0.74

## Discussion

The prevalence of dementia within the family medicine team where our study was conducted was 1.8%, which is substantially lower compared to epidemiological data from population-based studies, such as research in India where the age-standardized prevalence was 8.0%. This discrepancy and the relatively low rate at the primary care team level can be explained by several methodological and clinical factors. First and foremost, the relatively small sample size inherent to a single primary care practice panel reduces statistical power and limits the generalizability of this rate to the broader population. Primarily, our study lacks data on the mean age of the total registered population within the team, which significantly impacts the interpretation of this rate, given that the team provides care to patients across all age groups. Furthermore, primary healthcare settings frequently lack accessible diagnostic laboratory and neuroimaging resources required for the early verification of cognitive decline. Additionally, there is a noticeable risk of underdiagnosing dementia in its early stages, when subtle cognitive deficits are often overlooked or misattributed to non-specific neuropsychiatric symptoms, such as depression and anxiety, which are exceptionally common in this population [11].

The results obtained in our study suggest the potential significance of advanced age as a component of cognitive risk, aligning closely with prevailing epidemiological observations in the available literature. However, it is essential to interpret late-life risk profiles, such as physical inactivity and sensory decline, within the context of this significant age disparity between the cohorts (85.50 ± 4.83 vs. 80.34 ± 4.10 years; p < 0.05). Advancing age inherently acts as a major confounding variable, independently driving functional decline, sensory impairment, and physical inactivity regardless of cognitive status. Although certain epidemiological reports note variations in dementia prevalence between sexes, sex itself is not defined as an independent modifiable risk factor within the Lancet framework. Our findings support this assertion, as no statistically significant difference in sex distribution was found between the group without dementia and the comparative dementia group (p = 0.164). This observation is in full agreement with contemporary population studies which, after adjusting for age and socioeconomic factors, confirm the absence of significant sex and gender differences in the overall risk of developing dementia [12].

In evaluating early-life risk factors according to the Lancet framework, it is particularly notable that 55% (n = 11) of patients with dementia in our study had completed only primary education, while there were no participants with a higher (university) degree in this group [7]. Although the difference did not reach statistical significance in our sample, a clear trend toward a higher prevalence of lower educational attainment among affected individuals was observed. The aforementioned study also confirms that lower levels of formal education represent a significant independent risk factor for dementia development (HR = 1.29; p = 0.036). These findings lend support to the hypothesis that higher educational levels in early life stimulate cognitive reserve development, providing a protective effect in later years and delaying the clinical manifestation of cognitive decline [13].

Regarding BMI, our study demonstrated a statistically significant difference between the observed groups, with non-dementia patients exhibiting a significantly higher mean BMI compared to the dementia group. However, these findings require nuanced interpretation in geriatric populations. Although midlife obesity is established as a risk factor for cognitive decline in frameworks like the Lancet Commission [7], our cross-sectional evaluation captured participants in late life. Notably, while the dementia cohort displayed a relatively lower mean BMI than controls, a value of 26.05 kg/m² still falls within the overweight category rather than indicating clinical underweight. While late-life weight decline is frequently recognized as a prodromal feature or consequence of neurodegeneration, the cross-sectional nature of current measurements precludes confirming active weight loss or establishing direct causality.

An additional explanation for these findings is provided by a recent large-scale meta-analysis (encompassing over 4.2 million participants), which emphasizes that beyond static BMI values, body weight dynamics play a pivotal prognostic role. Specifically, increased intra-individual variability in body weight and BMI was significantly associated with a higher risk of incident dementia, regardless of subtype (Alzheimer's disease or vascular dementia), with variability remaining a significant predictor even after adjusting for baseline BMI [14].

In our study, a high prevalence of arterial hypertension was recorded across both groups, with a statistically significantly higher prevalence in participants without dementia. Although the Lancet Commission lists midlife hypertension as a key risk factor, the literature indicates that chronically elevated blood pressure is the primary driver of target organ damage, including the brain [7]. The higher rate observed in the non-dementia group may be attributed to higher BMI and more frequent routine check-ups in primary care, as well as the recognized clinical phenomenon in late-stage dementia where blood pressure often declines due to autonomic dysfunction or medication effects [15].

Regarding alcohol consumption, no patients in our sample reported consuming alcohol, classifying all participants as abstainers. Per the Lancet Commission recommendations, excessive midlife alcohol intake is a modifiable risk factor that directly contributes to neurotoxicity and elevates dementia risk [7]. However, interpreting the role of alcohol in older populations involves considerable complexity. A recent large international meta-analysis (n > 24,000) indicated that complete abstinence in late life was associated with a higher risk of developing dementia compared to light-to-moderate consumption, with risk showing no significant difference between lifelong abstainers and former drinkers. While these data suggest that light consumption may not exert harmful effects at the population level, the authors emphasize that neuroimaging (MRI) evidence continues to demonstrate that even low alcohol doses can adversely affect brain parenchyma and overall health [16]. In our setting, the absence of reported alcohol intake may also be attributed to sociodemographic and cultural characteristics of the population, alongside the tendency of older patients to underreport drinking habits; thus, complete abstinence reported in primary care should be interpreted cautiously and within the context of the patient's overall cardiovascular and neuropsychiatric profile.

Hearing impairment demonstrated universal prevalence across our entire study sample. Although this parameter lacked discriminative power between groups, it represents a crucial modifiable risk factor. Longitudinal studies and the Lancet Commission identify hearing loss as a robust, independent predictor of dementia that accelerates cognitive reserve depletion and social isolation [7]. Its universal presence in our primary care cohort highlights hearing impairment as a widespread yet under-addressed risk, suggesting that timely hearing correction in primary care could play a pivotal role in preventing cognitive decline [17].

With respect to smoking status, an insignificant number of smokers was recorded, precluding adequate statistical analysis or establishing a significant association between smoking and dementia risk. While general population and longitudinal studies clearly recognize current smoking as an independent factor significantly increasing dementia risk and mortality, this distribution in our work reflects the specificities of older primary care populations [18].

Diabetes mellitus is established as a major modifiable risk factor for late-life dementia. Our results demonstrated diabetes in approximately one-third of participants in both groups, with no statistically significant difference between them. Despite these findings, strict glycemic control and management of micro- and macrovascular complications remain imperative due to their documented adverse effects on cognitive function [19].

In our study, a high frequency of depression was recorded across both groups (n = 32; 64% in the non-dementia group vs. n = 11; 55% in the dementia group), with no statistically significant difference observed between them. Nevertheless, this high overall frequency merits special clinical consideration. In the broader literature, depression represents a key factor accompanying cognitive decline, with a reported prevalence of around 32% in individuals with mild cognitive impairment [20]. The substantial presence of depressive symptoms across both of our cohorts suggests that late-life depression may be frequently underestimated or undertreated in primary care settings. Consequently, these findings underscore the clinical necessity of implementing systematic depression screening for all patients aged 65 and older, irrespective of cognitive status, to enable timely intervention and potentially attenuate cognitive deterioration [20].

When analyzing other late-life modifiable risk factors, our sample revealed an exceptionally high, widespread prevalence of physical inactivity (n = 37; 74% in non-dementia vs. n = 18; 90% in dementia patients) and social isolation (n = 33; 66% vs. n = 12; 60%), while head trauma was present in a minimal percentage (n = 2; 4% vs. n = 1; 5%). No statistically significant differences were observed between groups for these parameters. These results indicate that such risk factors represent ubiquitous correlates of aging within the broader primary care population, independent of dementia status. In such homogeneous, older cohorts, their universal presence suggests they operate as part of a broader cumulative physiological burden, explaining the lack of individual group differences despite their well-established clinical significance in global literature [7].

The findings of this study should be interpreted in the context of several potential limitations. First, the research was conducted on a single primary care cohort (N = 70). Although a complete census approach was applied to capture all verified dementia cases within the panel and minimize selection bias, the small sample size limits the statistical power to detect significant differences for low-frequency risk factors, such as head trauma or active smoking. Furthermore, the baseline age gap between the cohorts represents a notable confounding factor; older age in the dementia group may independently accentuate the prevalence of sensory deficits and physical inactivity, masking or exaggerating true disease-specific associations. Second, the observational design precludes establishing direct cause-and-effect relationships between analyzed factors and dementia onset. A further notable limitation is that MMSE testing was not routinely performed in the non-dementia group, meaning the presence of unregistered, milder forms of cognitive impairment cannot be entirely ruled out within this cohort. In addition, hearing impairment assessment relied solely on historical and collateral anamnesis without objective audiometric testing, which may have impacted precision. Certain key factors in modern frameworks, such as air pollution, could not be evaluated due to the unavailability of local micro-level data. Finally, data collection via medical records carries inherent risks of information bias, particularly concerning retrospective assessments of the duration or severity of conditions such as late-life depression and social isolation.

## Conclusions

The results of our study, conducted in a primary healthcare setting, highlight the complex interplay and high prevalence of modifiable risk factors among the older population. Certain parameters, such as arterial hypertension, demonstrated widespread prevalence, while body mass index showed a statistically significant difference between the non-dementia and dementia groups. Other factors, including hearing impairment, depression, social isolation, and physical inactivity, were recorded in high proportions across both groups. The universal presence of these factors in primary practice suggests that they constitute a ubiquitous, cumulative burden within the older population. Although individual differences between groups for specific risks did not consistently reach statistical significance, these findings underscore the urgent need for early identification, multimodal prevention, and comprehensive management of modifiable risk factors at the primary care level to mitigate cognitive decline.
